# (−)-Epicatechin 3-O-β-d-allopyranoside prevent ovariectomy-induced bone loss in mice by suppressing RANKL-induced NF-κB and NFATc-1 signaling pathways

**DOI:** 10.1186/s12906-017-1737-9

**Published:** 2017-05-03

**Authors:** Hung-Bo Hsiao, Jin-Bin Wu, Wen-Chuan Lin

**Affiliations:** 1 Department of Life Sciences, National Chung Hsing University, Taichung, Taiwan; 2 School of Pharmacy, China Medical University, 91 Hsueh Shih Road, Taichung, 404 Taiwan, People’s Republic of China

**Keywords:** ECAP, *Davallia formosana* Hayata, Osteoclastogenesis, Osteoporosis, Osteoclast, RANKL

## Abstract

**Background:**

*Davallia formosana* Hayata is a herb that has been used in Chinese medicine to treat bone diseases, including arthritis, bone fractures and osteoporosis. The rhizome of *D. formosana* H. has been found to be rich in (−)-Epicatechin 3-O-β-d-allopyranoside (ECAP), which is considered to be the active component of the plant in terms of its antiosteoporotic effect. This study investigated the molecular mechanism of the antiosteoporotic property of ECAP isolated from the roots of *D. formosana* H. using both in vitro and in vivo models.

**Methods:**

We studied the effects of ECAP on the signaling pathways of the receptor activator of nuclear factor-κB ligand (RANKL)-stimulated osteoclastogenesis and ovariectomy-induced osteoporosis. In the in vitro study, the inhibitory action of ECAP on RANKL-induced osteoclastogenesis and the expression of osteoclast-related marker genes were investigated, and in the in vivo study, the effects of ECAP on bone were evaluated using ovariectomized (OVX) mice orally-administered ECAP for 4 weeks.

**Results:**

We demonstrated that ECAP dose-dependently inhibited RANKL- and nuclear factor of activated T-cells, and cytoplasmic 1 (NFATc-1)-induced osteoclastogenesis by RAW 264.7 cells, and reduced the extent of bone resorption. Furthermore, μCT images and TRAP staining showed that oral administration of ECAP to OVX mice prevented bone loss. ECAP administration also exerted recovery effects on serum C-terminal telopeptide of type I collagen and osteocalcin levels in OVX mice. In addition, we also found that MMP-9 expression was decreased in vivo and in vitro.

**Conclusions:**

Overall, our findings suggested that ECAP suppresses RANKL-induced osteoclastogenesis through NF-κB and NFATc-1 signaling pathways, and has the potential for use in osteoporosis treatment.

## Background

Disruption of the balance between bone resorption and new bone formation may lead to metabolic bone diseases, such as postmenopausal osteoporosis, autoimmune arthritis, and Paget’s disease [1]. Excessive bone resorption over bone formation results in total bone loss, thus leading to these diseases. Therefore, osteoclasts, the only bone cells capable of resorbing bone efficiently, are useful targets for the development of antiresorptive drugs for bone resorption diseases. Of the cytokines critical for osteoclastogenesis, macrophage colony-stimulating factor (M-CSF) and receptor activator of nuclear factor-κB ligand (RANKL) have been shown to be essential for osteoclast differentiation [2]. M-CSF is crucial factor for osteoclast precursor proliferation and survival, and is also associated with the process of induction of RANK expression in osteoclast precursors [3]. RANKL, a tumor necrosis factor (TNF) family cytokine, is able to stimulate the entire development processes of osteoclasts [4].

**Fig. 1:**
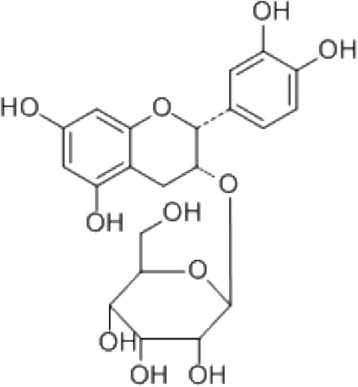
Structure of ECAP

We previously demonstrated that *Davallia formosana* Hayata extract (DFE) prevented bone loss in ovariectomized (OVX) rats, and also inhibited the differentiation of RAW 264.7 cells into osteoclasts [5]. *Davallia formosana* Hayata is a Taiwanese herb used as a substitute for the traditional Chinese herb Gu-Sui-Bu (*Drynaria fortunei*). It has been shown that (**−**)-epicatechin 3-O-β-d-allopyranoside (ECAP; Fig. 1) is the primary active compound of *Davallia formosana* Hayata ([5]). Based on the antiosteoclastogenesis-guided fractionation principle, we therefore further isolated the active component, ECAP, and validated its function in the RAW 264.7 cell differential system [5]. Moreover, recent study also showed that the ethanol extract of a fresh rhizome of *D. formosana* inhibited osteoclast differentiation through the inhibition of NF-κB activation [6]. Therefore, ECAP has potential as a new drug for metabolic bone diseases.

The NF-κB signaling pathway is essential in osteoclast formation, and there is already wide clinical application of ECAP; therefore, we hypothesized that ECAP could be a treatment option for osteoclast-related diseases. In this study, we aimed to investigate the potential therapeutic benefits of ECAP for the treatment of osteoclast-related diseases, and further examine the detailed underlying molecular mechanisms mediating the effects of ECAP on osteoclast formation and function.

## Methods

### ECAP preparation

Rhizomes of *Davallia formosana* H. were purchased from a local market in Taichung, Taiwan. The plants were identified in the Institute of Chinese Pharmaceutical Sciences, China Medical University, where a plant specimen was deposited (no. CMCP 1253). ECAP was isolated from fresh whole plants of *Davallia formosana* H. [5]. The identity and purity of ECAP (> 85%) was analyzed by HPLC as described in a previous study [5].

### Cell cultures

RAW 264.7 cells were cultured in α-MEM (Hyclone, Logan, UT, USA) supplemented with 10% FBS, 100 U/ml of penicillin, and 100 μg/ml of streptomycin. For osteoclast differentiation, RAW 264.7 cells, at 1 × 10^3^, were seeded in 24-well plates and treated human recombinant soluble receptor for the activation of NF-κB (RANKL, 50 ng/ml; PeproTech EC, London, UK) for 5 days. The culture medium was replaced every 3 days. 10, 50 and 100 μg/ml of ECAP were added to these cultures. After 3 days, the culture medium containing the relevant reagents (indicated above) was changed.

Osteoclast formation was measured using a TRAP staining kit as previously described [7]. Briefly, adherent cells were fixed with 10% formaldehyde in phosphate-buffered saline (PBS) for 3 min and then stained with naphthol AS-MX phosphate and tartrate solution for 1 h at 37 °C. Multinucleated TRAP-positive cells and bone marrow cells was detected using an MTS assay (CellTiter 96 Aqueous One Solution Cell Proliferation assay, Promega Corporation, Madison, WI, USA). The result was expressed in optical density units.

### Bone resorption

RAW 264.7 cells were suspended in α-MEM containing 10% FBS and plated at 1 × 10^4^ cells/well on Osteoclast Activity Substrate plates (OCT USA, Inc.) in the presence of 50 ng/ml of RANKL and incubated for 24 h. Subsequently, ECAP (10–100 μg/ml) was added to the cultures. Half of the medium was replaced with fresh medium every 2 days. After a 7-day culture, the plates were washed in 6% sodium hypochlorite solution to remove the cells. Images of resorbed areas on the plates were captured using a digital camera attached to an Olympus microscope and were analyzed using an automated software analysis program (Image-Pro Plus Version 5.1; Media Cybernetics, MD, USA).

#### RT-PCR analysis

RAW 264.7 cells were suspended in α-MEM containing 10% FBS in the presence of 50 ng/ml or RANKL and incubated for 1 h. ECAP (10–100 μg/ml) was added to the cultures. Mouse cathepsin K (CAK), MMP-9, carbonic anhydrase II (CAII), TRAP of treatment with RANKL in the absence or presence of ECAP (10–100 μg/ml), total RNA was extracted using TRIzol reagent (Invitrogen, Carlsbad, CA, USA) according to the manufacturer’s instructions. The PCR primer sequences for mouse CAK, MMP-9, CAII, TRAP, and GAPDH were as follows: CAK, 5′-CTGCCCATAACCTGGAGG-3′ (sense) and 5′-GCCCTGGTTCTTGACTGG-3′ (antisense); MMP-9, 5′-GGTCTAGGCCCAGAGGTA-3′ (sense) and 5′-GGTCGTAGGTCACGTAGC-3′ (antisense); CAII, 5′-CCCACCACTGGG GATACA-3′ (sense) and 5′- AGGGGTCCTCC TTTCAGC-3′ (antisense); TRAP, 5′-GAACCGTGCAGACGATGG-3′ (sense) and 5′-GGAAGTT CCAGCGCTTGG-3′ (antisense); and GAPDH, 5′-CTTCATTGACC TCAACTACATGGTCTA-3′ (sense) and 5′-GATGACAAGCTTCCCATTCTCAG-3′ (antisense). The expected sizes of PCR products were 230 bp, 310 bp, 247 bp, 231 bp, and 99 bp for CAK, MMP-9, CAII, TRAP, and GAPDH, respectively. All primer sets were designed using NCBI Primer-BLAST.

### Gelatin zymography

The enzymatic activity of MMP-9 was determined using gelatin zymography. Briefly, RAW 264.7 cells were seeded and allowed to grow to confluence for 24 h, and then maintained in 1% FBS-supplemented medium with different concentrations of ECAP (10, 50, and 100 μg/ml) in the absence or presence of RANKL (50 ng/ml). Subsequently, the culture medium was collected and centrifuged at 14,000 rpm for 5 min at 4 °C to remove cell debris. Protein content was measured using the Bradford method. The culture medium was mixed with 4× nonreducing sample buffer (4:1, *v*/v) and subjected to electrophoresis in a 10% polyacrylamide gel containing 0.1% (*w*/*v*) gelatin. The gel was washed with washing buffer containing 3% Triton X-100 and incubated at 37 °C for 16 h in 50 mM Tris (pH 7.5), 200 mM NaCl, 5 mM CaCl_2_, and 0.2% (*w*/*v*) NaN_3_. The gel was stained with 0.2% (*w*/*v*) Coomassie Brilliant Blue R-250 in 50% (*v*/v) methanol and 10% (*v*/v) acetic acid.

### Western blotting

To determine the levels of protein expression in the cytoplasm or the nucleus, we prepared extracts [7] from RANKL-treated cells and fractionated them by SDS-PAGE.

After electrophoresis, the proteins were electrotransferred to nitrocellulose membranes, blotted with each Ab. By using the enhanced chemiluminescence detection system (Amersham Biosciences, Inc., Piscataway, NJ, USA), specific bands were detected and the membrane was exposed to an X-ray film. For densitometry analysis, the OD was measured on the inverted digital images using AlphaEase (Alpha Innotech Corporation, San Leandro, CA, USA).

### Animal experiments

Mice (18–20 g, ~7 weeks old) were ovariectomized (OVX) and sham-operated (Sham). For the surgery, Female ICR mice were anesthetized with pentobarbital sodium. After 3 days of recovery from surgery, OVX mice were randomly divided into 4 groups and orally treated with H_2_O, ECAP (50 or 100 mg/kg/day), or alendronate (2.5 mg/kg 3 times a week; Sigma–Aldrich) for 4 weeks. The Sham group was orally treated with H_2_O as described previously [7]. The experimental animals were housed in an air-conditioned room at 22–25 °C under a 12-h light/dark cycle. All animals were treated in accordance with the Institutional Animal Care and Use Committee (IACUC) of China Medical University, and the study protocol was approved by the ethics committee of the China Medical University, Taichung, Taiwan.

Serum osteocalcin (OCN) levels were measured using clinical kits (Nordic Bioscience Diagnostics, Herlev Hovedgade, Denmark). Serum C-terminal telopeptide of type I collagen (CTx) levels were measured using a mouse-specific ELISA assay kit according to the manufacturer’s protocols (Nordic Bioscience Diagnostics).

The trabecular bone microarchitecture of the distal left femoral metaphysis was determined using a microcomputed tomography (micro-CT) scanner (SkyScan 1076, Kontizh, Belgium), with an isotropic resolution of 18 μm in all 3 spatial dimensions. Briefly, micro-CT analysis was performed using bone-related parameters, including Trabecular bone volume (%), number of trabeculae (No./mm), thickness of the trabeculae (μm), and separation of trabeculae (μm) which are minimal set of variables that should be investigated for trabecular bone regions [7].

The left femur was removed, fixed with 4% neutral-buffered paraformaldehyde in PBS (pH 7.4) for 48 h, and decalcified in 10% ethylenediamine tetraacetic acid solution (pH 7.4) at 4 °C for 4 weeks. After decalcification, each bone sample was examined by histological analysis including hematoxylin and eosin (H&E) and TRAP staining as previously described [7].

To study the ECAP-associated mechanisms on OVX-induced osteoporosis in mice, total RNA of the right tibiae was extracted for RT-PCR analysis. The expression levels of *MMP-9* and *TRAP* were normalized to those of *GAPDH* mRNA in the same tissue. PCR products were separated on a 2% agarose gel and recorded on a Polaroid film, and the intensity of the band was quantified using a densitometer. The mean ratio of each group was calculated as the average for 8 mice.

#### Statistical analysis

All results are expressed as mean ± SD. All experimental data were analyzed using one-way analysis of variance with Dunnett’s test. Results with *P* < 0.05 were considered statistically significant.

## Results

### Effects of ECAP on osteoclastogenesis and activity in RAW 264.7 cells

**Fig. 2:**
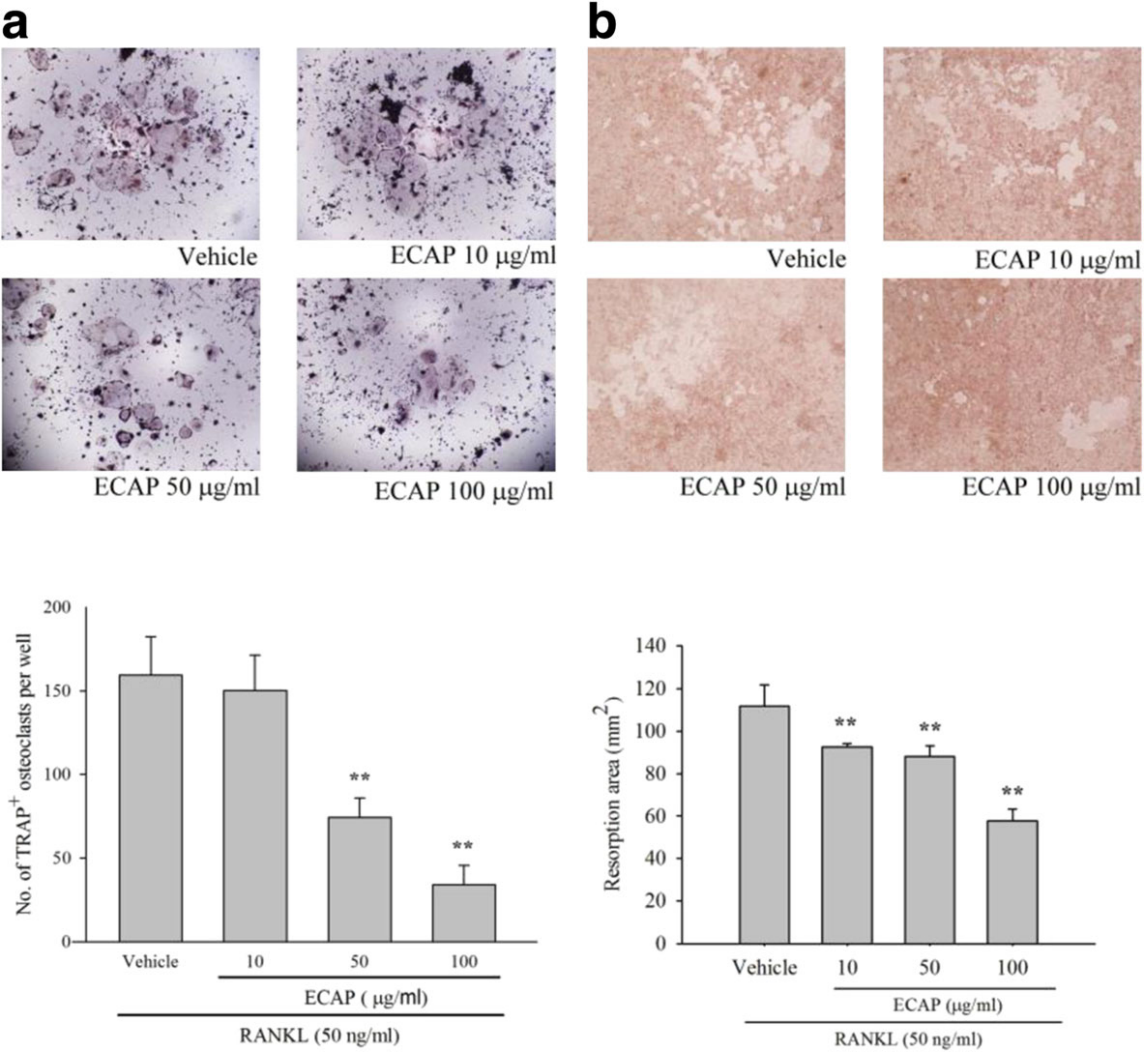
Effects of ECAP on RANKL-induced osteoclastogenesis activity. **a** RAW 264.7 cells were cultured for 5 days in the presence of RANKL (50 ng/ml) with the vehicle (H_2_O) or ECAP. Multinucleated osteoclasts were visualized by TRAP staining. TRAP-positive cells were photographed under a light microscope. Multinucleated TRAP-positive cells with ≥3 nuclei were defined as osteoclasts. **b** RAW 264.7 cells were cultured on Osteoclast Activity Substrate plates in the presence of RANKL (50 ng/ml) with the vehicle (H_2_O) or ECAP for 7 days. After a 7-day culture, adherent cells were removed and photographed under a light microscope. Each value represents the mean ± SD. (*n* = 3). **P < 0.01 compared with RANKL alone

Using a tetrazolium compound MTS assay, we found that treatment of RAW 264.7 cells with ECAP (10–100 μg/ml) for 3 days did not affect cell viability (data not shown). In addition, ECAP exhibited a dose-dependent effect on the inhibition of RANKL-induced osteoclast differentiation in RAW 264.7 cells (Fig. 2a). Moreover, ECAP reduced osteoclast formation by 93%, 50% (*p* < 0.05) and 71% (*p* < 0.05) at 10, 50, and 100 μg/ml, respectively.

We then studied bone resorption by mature osteoclasts. RAW 264.7 cells were seeded on bone slices and the culture was stimulated with RANKL in the presence or absence of ECAP. With the RANKL-stimulated cells, many pits were formed on the bone slices (Fig. 2b), indicating that the bone resorption activity of RANKL-stimulated cells transformed the cells into functionally-active osteoclasts. Compared with RANKL treatment alone, ECAP treatment (10–100 μg/ml) significantly reduced the number and area of resorption pits on the bone slices. ECAP inhibited osteoclast resorption by 20% (10 μg/ml; *p* < 0.01), 24% (50 μg/ml; *p* < 0.01), and 67% (100 μg/ml; *p* < 0.01).

### ECAP inhibited MMP-9 expression in RANKL-stimulated RAW 264.7 cells

**Fig. 3:**
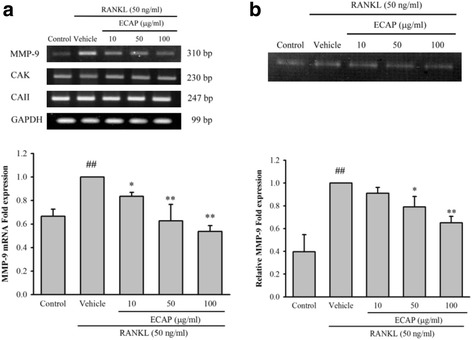
Effects of ECAP on mRNA and protein expression of steoclastogenesis-related genes. **a** RAW 264.7 cells were cultured in the presence of RANKL (50 ng/ml) with the vehicle (H_2_O) or ECAP. After 24 h, total RNA was then isolated using TRIzol reagent, and mRNA expression levels were evaluated by RT-PCR. Glyceraldehyde- 3-phosphate dehydrogenase (GAPDH) was used as the internal control. **b** RAW 264.7 cells were cultured in the presence of RANKL (50 ng/ml) with the vehicle (H_2_O) or ECAP. After 24 h, the culture medium was collected and analyzed by gelatin zymography. Each value represents the mean ± SD. (*n* = 3). ## *P* < 0.01 compared with control; *P < 0.05, **P < 0.01 compared with RANKL alone

By performing RT-PCR, we examined the effects of ECAP on the expressions of osteoclastogenesis-associated genes. RT-PCR analysis of total RNA isolated from RANKL-stimulated RAW 264.7 cells demonstrated that RANKL stimulation significantly increased the MMP-9 mRNA level (Fig. 3a). On the other hand, in terms of the effects on osteoclastogenesis-related genes, ECAP reduced the MMP-9 mRNA level in the presence of RANKL, but did not alter the CAK and CA II mRNA levels (Fig. 3a). As MMP-9 is considered essential for mediating the mobility of preosteoclasts and osteoclasts, we examined whether ECAP could reduce RANKL-induced MMP-9 activation. The results showed that ECAP inhibited the MMP-9 expression level in RANKL-stimulated RAW 264.7 cells (Fig. 3b).

### ECAP suppresses multiple pathways in RANKL-stimulated RAW 264.7 cells

**Fig. 4:**
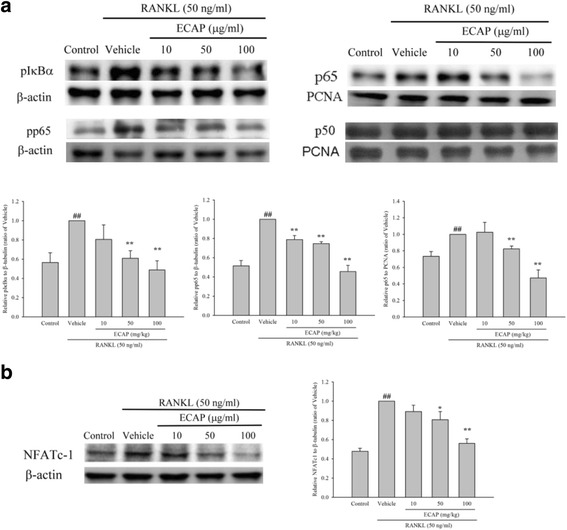
Effects of ECAP on RANKL-induced NF-κB and NFATc1 activation. **a** RAW 264.7 cells were pre-incubated for 1 h with indicated concentrations of ECAP and then activated for 1 h with RANKL (50 ng/ml). Cytoplasm fractions were obtained for the detection of phospho-IκBα, phospho-p65 and β-actin levels. Nuclear fractions were obtained for the detection of p65, p50 and PCNA levels. **b** RAW 264.7 cells were pre-incubated for 1 h with indicated concentrations of ECAP and then activated for 24 h with RANKL (50 ng/ml). The cell lysates were obtained for the detection of NFATc1 levels. Each value represents the mean ± SD. (*n* = 3). ## *P* < 0.01 compared with control; *P < 0.05, **P < 0.01 compared with RANKL alone

RAW 264.7 cells were treated with RANKL in the presence or absence of ECAP for 120 min. According to western blotting results, treatment with RANKL for 60 min increased the cytoplasmic protein expression levels of *p*-IκBα and *p*-p65 (Fig. 4a). The expression levels of *p*-IκBα and *p*-p65 in the RANKL-stimulated RAW 264.7 cells (RANKL group) were increased to 162% and 212%, respectively; these levels were significantly greater than those in the control group. Although ECAP treatment did not affect the total IκBα protein expression, it did however reduce the *p*-IκBα expression by 35% (50 μg/ml) and 52% (100 μg/ml), and reduced the *p*-p65 expression by 18% (10 μg/ml), 22% (50 μg/ml), and 55% (100 μg/ml) (Fig. 4a). We then determined the expression levels of p65 in the cytoplasm and the nucleus. The expression level of p65 in the nuclei of the cells of the RANKL group was 142% of that in the nuclei of the cells in the control group; in addition, ECAP pretreatment reduced the p65 expression by 20% (50 μg/ml) and 55% (100 μg/ml) (Fig. 4a).

When RAW 264.7 cells were incubated with RANKL with or without ECAP for 120 min, western blotting analysis showed an increased NFATc-1 protein expression level in the cells treated with RANKL for 24 h (Fig. 4b). The NFATc-1 protein level in the RANKL group was 203% of that in the control group, and ECAP pretreatment reduced the NFATc-1 protein expression by 20% (50 μg/ml) and 43% (100 μg/ml).

### Effects of ECAP on bone loss and osteoclast activity in OVX mice

The trabecular bone volume, trabecular number, and trabecular thickness were lower in the OVX mice than in the sham-operated mice (Table 1), and the OVX mice also showed increased trabecular separation as compared with the sham-operated mice. Treatment of the OVX mice with ECAP (50 or 100 mg/kg/day) or alendronate prevented ovariectomy-induced reduction in the trabecular bone volume, trabecular number, and trabecular thickness, and prohibited ovariectomy-induced increased trabecular separation. The expression levels of the trabecular bone volume, trabecular number, and trabecular thickness in the OVX mice were 69.8%, 83%, and 87% respectively; these values were lower than those in the sham-operated mice. The expression level of trabecular separation in the OVX mice was 147%, which was greater than that in the sham-operated mice. Trabecular area (%), trabecular number, trabecular thickness and trabecular separation markedly improved in the ECAP-treated OVX mice as compared with the ECAP vehicle-treated OVX mice. ECAP pretreatment increased the trabecular area (%) by 118% (50 mg/kg) and 119% (100 mg/kg), the trabecular number by 113% (50 mg/kg) and 113% (100 mg/kg), and the trabecular thickness by 16% (100 mg/kg), in addition to reducing trabecular separation by 80% (100 mg/kg) (Table 1).

**Table 1:** Effects of ECAP on the percentage of trabecular bone, number of trabeculae, thickness of the trabeculae and separation of trabeculae of the distal femoral metaphysis in OVX mice by microtomography analysis

Drugs	Doses (mg/kg)	Trabecular bone volume (%)	Number of trabeculae (No./mm)	Thickness of the trabeculae (μm)	Separation of trabeculae (μm)
Sham	0	29.5 ± 1.9	2.8 ± 0.3	91.8 ± 2.9	212.0 ± 30.4
OVX + Vehicle	0	20.6 ± 1.6##	2.3 ± 0.2##	80.1 ± 3.9##	312.2 ± 25.6##
OVX + ECAP	50	24.3 ± 2.2*	2.6 ± 0.2*	84.4 ± 4.1	275.5 ± 15.4
OVX + ECAP	100	24.5 ± 0.8**	2.6 ± 0.1*	85.4 ± 1.8*	250.6 ± 25.8**
OVX + Alendronate	2.5	26.2 ± 2.0**	2.7 ± 0.2**	86.8 ± 4.3**	227.2 ± 34.1**

##*P* < 0.01 compared with shame group; *P < 0.05, **P < 0.01 compared with OVX + Vehicle group

**Fig. 5:**
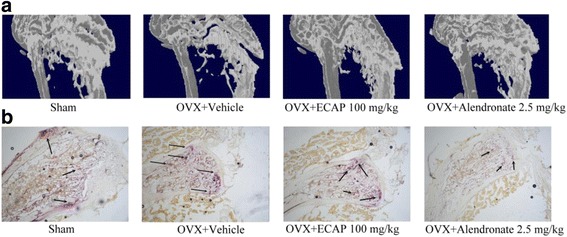
ECAP inhibits osteoporosis in OVX mice. **a** The distal left femoral metaphysis was scanned using a micro-CT after 4 weeks in OVX mice. **b** The left femora were fixed, decalcified, embedded, and sectioned. Sections were stained with TRAP after 4 weeks in OVX mice

These results were further supported by μCT images and TRAP staining of decalcified bone sections. μCT images of the distal left femoral metaphysis revealed that ECAP inhibited ovariectomy-induced bone loss (Fig. 5a). Compared with the vehicle-treated OVX mice, the ECAP-treated mice had a decreased number of TRAP-positive multinucleated cells at the growth plates of the left femur (Fig. 5b).

### Serum C-terminal telopeptide of type I collagen (CTx) and osteocalcin (OCN) levels

Ovariectomy caused significant increases in the serum CTx and OCN levels in the OVX mice (Table 2). Treatment with ECAP (50 or 100 mg/kg) or alendronate suppressed the increases in serum CTx and OCN levels in the OVX mice. The expression levels of CTx and OCN in the OVX mice were 246% and 126%, respectively; these levels were higher than those in the sham-operated mice. ECAP pretreatment reduced the CTx level by 85% (50 mg/kg) and 81% (100 mg/kg) and the OCN level by 88% (50 mg/kg) and 88% (100 mg/kg).

**Table 2:** Effects of ECAP on plasma CTx and Osteocalcin contentrations in OVX mice

Drugs	Doses (mg/kg)	CTx(ng/ml)	Osteocalcin (ng/ml)
Sham	0	5.6 ± 1.2	27.9 ± 1.1
OVX + Vehicle	0	13.8 ± 1.1##	35.4 ± 1.3##
OVX + ECAP	50	11.8 ± 1.0*	31.3 ± 2.0**
OVX + ECAP	100	11.3 ± 1.1**	31.4 ± 1.4**
OVX + Alendronate	2.5	8.9 ± 1.6**	30.7 ± 1.3**

##*P* < 0.01 compared with shame group; *P < 0.05, **P < 0.01 compared with OVX + Vehicle group

### RT-PCR analysis of tibial mRNA expression in OVX mice

**Fig. 6:**
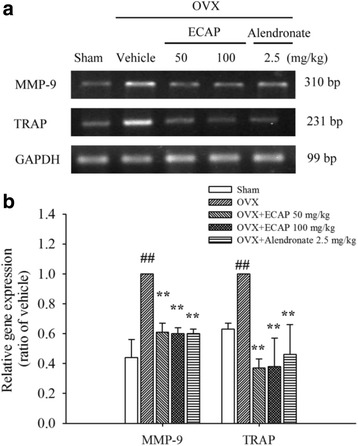
The expression of MMP-9 and TRAP RT-PCR on the metaphysis of the right tibiae in OVX mice. **a** Fragments of MMP-9, TRAP and GAPDH were amplified by RT-PCR. **b** The expression levels of MMP-9 and TRAP mRNA were measured and quantified densitometrically after 4 weeks in OVX mice. Each value represents the mean ± SD. (*n* = 8). ## *P* < 0.01 compared with shame group; *P < 0.05, **P < 0.01 compared with OVX + Vehicle group

The fragments shown in Fig. 6 reflect pooled data for 8 samples. RT-PCR analysis of the tibial samples shown in Fig. 6 demonstrated that the expressions of *MMP-9* and *TRAP* were 250% (*p* < 0.01) and 167% (*p* < 0.05) higher in the OVX mice than in the sham-operated mice. ECAP treatment led to 37% (50 mg/kg) and 42% (100 mg/kg, *p* < 0.01) decreases in MMP-9 expression and 64% (50 mg/kg, *p* < 0.01) and 60% (100 mg/kg, *p* < 0.01) decreases in TRAP expression. Alendronate treatment led to a 37% (*p* < 0.01) decrease in MMP-9 expression and a 50% (*p* < 0.01) decrease in TRAP expression.

## Discussion

Bone metabolism is regulated by a balance between new bone formation by osteoblasts and old bone resorption by osteoclasts. Excessive RANKL signaling causes enhanced osteoclast formation and bone resorption. Downregulating RANKL expression or its downstream signals may be a valuable approach for the treatment of pathological bone loss. In this study, we report for the first time the antiosteoclastogenic activity of ECAP, the primary active compound of *D. formosana* H., a Taiwanese herb that has often been used as a substitute for the traditional Chinese herb Gu-Sui-Bu (*D. fortunei*). We demonstrated that ECAP effectively suppressed RANKL-induced osteoclast differentiation and formation in vitro. At the molecular level, ECAP suppressed RANKL-induced expression of NF-κB, and NFATc1 expression was dramatically downregulated. Finally, ECAP inhibited the expressions of mature osteoclast-related marker genes such as MMP-9 and TRAP. Therefore, we demonstrated that ECAP also attenuated ovariectomy-induced osteoclastogenesis and prevented bone loss in vivo.

Osteoclasts, unique large multinucleated cells, play an important role in bone homeostasis, as they have the capacity to enhance bone resorption [8]. Increased RANKL signaling causes enhanced osteoclast formation and bone resorption. In general, two essential steps contribute to osteoclastogenesis: 1) commencing progenitor cells to osteoclast precursors, which involves activating osteoclast marker genes such as TRAP; and 2) forming multinucleated osteoclasts by merging the TRAP-positive mononuclear cells. In this study, we used a homogeneous, murine macrophage RAW 264.7 cell line to assess the direct effects of ECAP on RANKL-induced osteoclastogenesis. However, as this system did not contain any osteoblasts/bone marrow stromal cells or cytokine-like M-CSF, we were able to focus on RANKL signaling in preosteoclast cells. We previously demonstrated that *D. formosana* H. extract (DFE) reduced bone loss in OVX rats [5]. In this study, we isolated ECAP, the primary active compound of *D. formosana* H., and further demonstrated that ECAP suppressed RANKL-stimulated osteoclastogenesis in RAW 264.7 cells. In addition, the bone resorption assay results revealed that ECAP inhibited the bone resorption activity of mature osteoclasts. We then examined the effects of ECAP on mRNA expressions of osteoclastogenesis-related genes (MMP-9, CAK, and CAII), and observed that ECAP reduced RANKL-induced MMP-9 mRNA expression, but did not affect CAK or CAII mRNA expressions. These results suggested that the inhibitory effect of ECAP on osteoclastogenesis is attributable not only to its inhibitory effect on osteoclast differentiation, but also to the bone resorption activity of mature osteoclasts.

Gaining a greater understanding of the cellular and molecular mechanisms by which ECAP inhibits osteoclast differentiation might provide valuable information for the treatment of osteolysis. Several transcription factors, including PU.1, microphthalmia-associated transcription factor, NF-κB, c-Fos, and NFATc-1, have been shown to play a role in osteoclast differentiation from its precursors [8], whereas NF-κB, c-Fos, and NFATc1 function downstream of RANKL signaling for osteoclast differentiation. The RANKL receptor, RANK, lacks intrinsic enzymatic activity in its intracellular domain and activates NF-κB and MAPKs, including JNK and p38, in RANKL-induced osteoclastogenesis [9, 10]. In the canonical NF-κB pathway, RANK ligation activates the inhibitor of the IκB kinase (IKK) complex, which phosphorylates NF-κB-associated IκBα, resulting in its ubiquitination and proteosomal degradation [11]. NFATc1 is an NFAT family member that is activated by the Ca^2+^/calmodulin-regulated phosphatase calcineurin [12]. In osteoclast precursors, calcium signaling activates the existing NFATc1, and an AP-1 complex containing c-Fos may cooperate with NFATc-1 to trigger NFATc-1 amplification. Upon activation, NFATc-1 proteins are dephosphorylated by calcineurin and then translocated from the cytoplasm to the nucleus, where they regulate transcription of osteoclast-specific genes such as TRAP, CAK, and MMP-9 at the terminal differentiation stage of osteoclasts [13, 14]. Previous studies have shown that RANKL-induced activation of the NF-κB pathway plays a pivotal role in osteoclastogenesis [15, 16]. In addition, genetic and pharmacological study findings have underscored the importance of the NF-κB pathway in osteoclastogenesis [17–20]. In this study, we demonstrated that ECAP suppressed RANKL-induced activation of the NF-κB pathway, as demonstrated by the inhibition of phosphorylation and degradation of IκBα, inhibition of phosphorylation and nuclear translocation, and DNA-binding activity of NF-κB. In addition, studies have shown that NFATc1 is a master regulator in osteoclastogenesis [21, 22]. Furthermore, NF-κB and AP-1 can bind to the NFATc1 promoter and regulate NFATc1 expression, and NF-κB is also known to initially induce NFATc-1 expression at the early stage. Our data showed that ECAP inhibited osteoclastogenesis at the early stage, suppressed NF-κB activation, and inhibited NFATc-1 expression. Thus, our results strongly suggested that ECAP inhibits osteoclastogenesis by altering NF-κB activation. This finding is consistent with the observation that NF-κB downregulation reduces NFATc-1 expression [16, 23, 24].

**Fig. 7:**
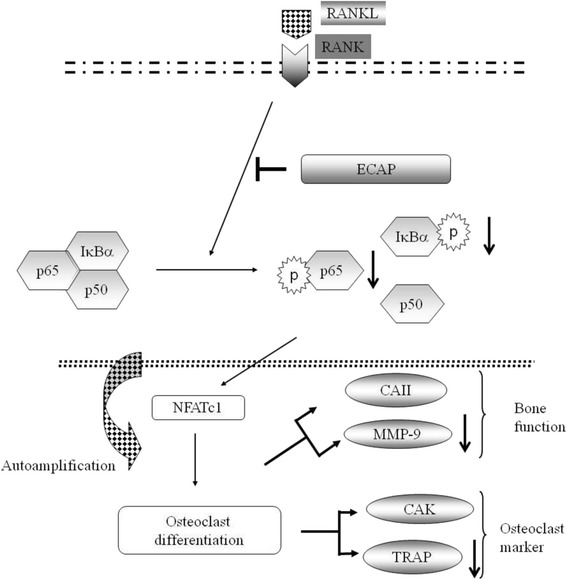
Proposed intracellular actions of ECAP to suppress RANKL-induced osteoclastogenesis in RAW264.7 cells. ECAP, (−)-Epicatechin 3-O-β-d-allopyranoside; CAII, carbonic anhydrase II; MMP-9, matrix metalloproteinase-9; CAK, cathepsin K; TRAP, tartrate-resistant acid phosphatase; NFATc1, nuclear factor of activated T cells c1; RANKL, receptor activator of nuclear factor kB (NF-kB) ligand

Bone homeostasis is a delicate process that relies on an adequate balance between bone resorption by osteoclasts and bone formation by osteoblasts [25]. In our previous study, we showed that DFE administration can prevent osteoporosis in OVX rats [5]. ECAP is the main active compound of *D. formosana*, and the amount of ECAP in DFE was found to be 3.2%*.* Bisphosphonate therapies are a class of drug used in prevention and cure of bone diseases like osteoporosis and associated with increased risk of cancer or osteonecrosis, respectively. In the present study, we aimed to evaluate the effect of ECAP in terms of a potential treatment for postmenopausal osteoporosis; therefore, we used an OVX mouse model to observe bone formation and bone resorption. We demonstrated that ECAP at 2 doses (50 or 100 mg/kg/day) could ameliorate OVX-induced bone loss in the mouse model. Quantitative microcomputed tomography analysis of the trabecular bone microarchitecture showed that ovariectomy caused a marked decrease in the trabecular bone volume per tissue volume, trabecular thickness, and trabecular number, and increased the trabecular separation. Oral administration of ECAP significantly attenuated RANKL-induced changes in the trabecular area (%) and the trabecular number. We then investigated changes in serum CTx and OCN levels, which are used as markers of bone resorption, and bone formation, respectively [26]. ECAP markedly inhibited ovariectomy-induced increases in serum CTx and OCN levels, therefore exerting a protective effect against bone destruction by suppressing bone resorption through the inhibition of osteoclast differentiation. Our results clearly showed that ECAP attenuates RANKL-induced osteoclast differentiation and bone destruction in vivo. TRAP activity is a marker often used for identifying osteoclasts [27], and MMP-9 is required for osteoclast migration and resorption [28]. In this study, ECAP administration inhibited the mRNA expressions of femoral TRAP and MMP-9 (Fig. 7).

## Conclusion

In summary, our results showed that ECAP has antiosteoclastogenic potential by altering NF-κB and NFAcT-1 activation. In addition, ECAP suppresses the bone resorption activity of mature osteoclasts by regulating the expression of osteoclast resorption-related genes. Moreover, ECAP decreases ovariectomy-induced osteoporosis in mice. Thus, our results strongly suggested that ECAP warrants evaluation as a potential treatment for osteoporosis.

## Abbreviations

CTx: C-Terminal telopeptide of type I collagen
ECAP: (−)-Epicatechin 3-O-β-d-allopyranoside
M-CSF: Macrophage colony-stimulating factor
MMP-9: Matrix metalloproteinase-9
NFATc-1: Nuclear factor of activated T-cells, cytoplasmic 1
OCN: Osteocalcin
OVX: Ovariectomized
RANKL: Receptor activator of nuclear factor-κB ligand
TRAP: Tartrate-resistant acid phosphatase
